# Secondary Lip Flow in a Cyclone Separator

**DOI:** 10.1007/s10494-023-00395-5

**Published:** 2023-01-24

**Authors:** Dzmitry Misiulia, Göran Lidén, Sergiy Antonyuk

**Affiliations:** 1 Institute of Particle Process Engineering, University of Kaiserslautern-Landau, Gottlieb-Daimler-Straße 44, 67663 Kaiserslautern, Rhineland-Palatinate Germany; 2 Department of Environmental Science, Stockholm University, Svante Arrhenius väg 8, 10691 Stockholm, Stockholm County Sweden

**Keywords:** Cyclone, Secondary flow, Lip flow, Large eddy simulation

## Abstract

Three secondary flows, namely the inward radial flow along the cyclone lid, the downward axial flow along the external surface of the vortex finder, and the radial inward flow below the vortex finder (lip flow) have been studied at a wide range of flow rate 0.22–7.54 LPM using the LES simulations. To evaluate these flows the corresponding methods were originally proposed. The highly significant effect of the Reynolds number on these secondary flows has been described by equations. The main finding is that the magnitude of all secondary flows decrease with increasing Reynolds number. The secondary inward radial flow along the cyclone lid is not constant and reaches its maximum value at the central radial position between the vortex finder external wall and the cyclone wall. The secondary downward axial flow along the external surface of the vortex finder significantly increases at the lowest part of the vortex finder and it is much larger than the secondary flow along the cyclone lid. The lip flow is much larger than the secondary inward radial flow along the cyclone lid, which was assumed in cyclone models to be equal to the lip flow, and the ratio of these two secondary flows is practically independent of the Reynolds number.

## Introduction

Cyclone separators have been widely used in various industrial and domestic applications, where there is a need in particle separation from the carrier gas. They are applied in different sizes, from several millimetres in diameter (small-scale cyclones) up to several meters in diameter (industrial-scale or large-scale cyclones). Large-scale cyclones are mostly utilised in industrial applications whereas small-scale cyclone are mainly used in vacuum cleaners, pre-filtration systems for automobile ventilation, internal combustion engines, and increasingly in aerosol sampling. Recently it was reported (Rahmani et al. 2020) that cyclone samplers showed suitable performance for trapping SARS-CoV viruses in air. Guo et al. (2020) identified SARS-CoV-2 in indoor air of hospital using SASS 2300 wetted wall cyclone sampler at a rather high sampling flow rate of 300 LPM (litre per minute), whereas (Chia et al. 2020) did it with a cyclone bioaerosol sampler at a much lower flow rate of 3.5 LPM.

Since last century improvement/optimisation of a cyclone geometry (inlet, roof (lid), vortex finder, barrel, cone, dust hopper, dust discharge opening) has been the target of numerous investigations. However only a few efforts were performed to study secondary flows in cyclones.

**Fig. 1:**
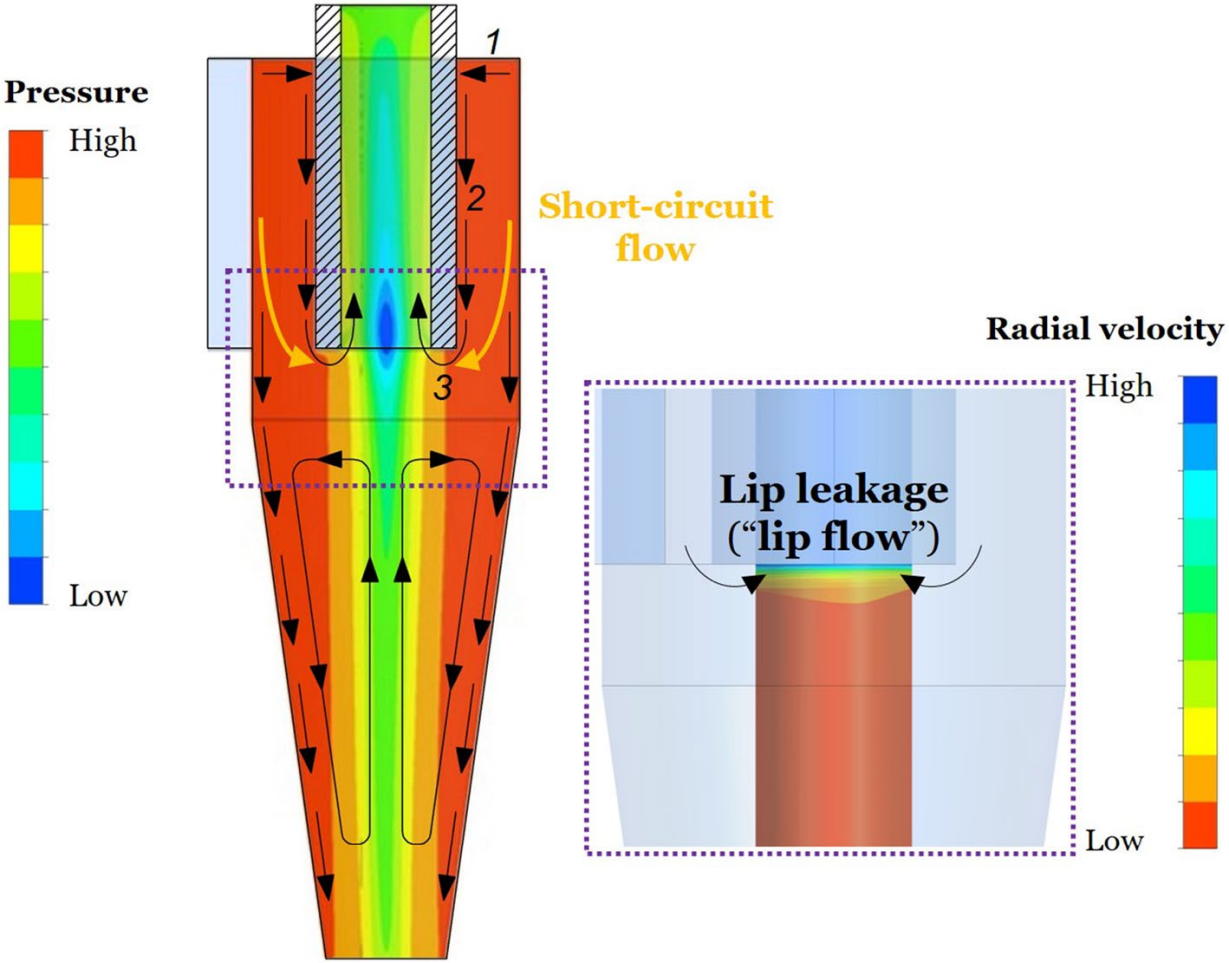
Secondary flows in a cyclone: *1*—inward radial flow along the cyclone lid, *2*—downward axial flow along the external surface of the vortex finder, *3*—inward radial flow below vortex finder (the lip flow). (The figure is simplified and only shows the general outline of the secondary flows)

Secondary flows along the walls are an inherent part of the flow field in a cyclone separator. They are created because of the pressure gradients caused by the primary, swirling motion of the gas (Fig. 1). Namely, large pressure is created on the walls while the vortex core region is characterised by low pressure. The inward flow along the cyclone lid drives a downward flow along the outer wall of the vortex finder. This contributes to the high inwardly directed radial velocities just below the vortex finder that is referred to as “lip leakage” or “lip flow”. In addition to these secondary flows along the walls, there is experimental evidence (as reported by Hoffmann and Stein (2008)) that a “swiss roll” type of secondary flow pattern exists in the cyclone cone, which can cause particles to recirculate in the cyclone.

This study focuses on the secondary flow along the cyclone lid and the vortex finder which contributes to the lip flow. The particles, captured by this secondary flow, are likely to leave the cyclone without being separated. Therefore, a lip flow can significantly affect the separation efficiency.

Ebert (1967) was the first who determined the thickness of the turbulent boundary layer in a cyclone and proposed an equation for calculation the secondary flow along the lid. He found that the thickness of the turbulent boundary layer is not constant along the radius and depends on the tangential velocity profile, which is characterised by the power factor in the "loss-free vortex".

Later, Trefz (1992) performed a comprehensive study of the secondary flow along the lid in an 800 mm diameter cyclone. He measured the radial and tangential velocities in the turbulent boundary layer at four different circumferential angles and different radii along the cyclone lid by the usage of a three orifice probe. Then he determined the secondary volumetric flow rate by integration of the radial velocity profile over the boundary layer thickness using the approximation function. Trefz found that the lip flow decreases with particle loading (the lip flow reduced from 17% for the pure air down to below 10% at high particle loading) and the average lip flow is 10–15%. That’s why, probably, a value of 10% is employed in the model most widely used by engineers, Muschelknautz’ model (Muschelknautz 2019), which assumes that industrial cyclones operate at rather high particle concentrations. According to the Muchelknautz model, the lip flow is assumed constant and independent of operational parameters and cyclone design.

Hoffmann (1998) also verified the existence of the particle-laden boundary layer along the cyclone lid (top cover). He measured flow velocities in the upper part of the cyclone employing two different tools, pressure probes and Laser Doppler Anemometry (LDA), and found that the pressure probes (they were also used by Trefz (1992)) overestimated the magnitude of the boundary flow, especially close to the cyclone wall. Hoffmann (1998) verified the overestimation of the pressure probes with experiments in a wind tunnel.

The main aim of this study is to address the questions:

- Does the lip flow depend on the Reynolds number?
- Does the lip flow equal the secondary radial inward flow along the lid?

In order to answer these questions, Computational Fluid Dynamics (CFD) was employed to investigate the following secondary flows:

1. Radial inward flow along the cyclone lid (marked as *1* in Fig. 1);
2. Axial downward flow along the external surface of the vortex finder (marked as *2* in Fig. 1);
3. Radial inward flow below the vortex finder (marked as *3* in Fig. 1). The lip flow most probably consists not only of the secondary axial flow along the external surface of the vortex finder, but the short-circuit flow as well.

## CFD Modelling

The particle-laden flow in a cyclone was modelled with the Euler-Lagrange approach where the continuous phase (air flow) was treated in a Eulerian manner, whereas the dispersed phase (particles) was treated in a Lagrangian method.

### LES Governing Equations for Air Flow in a Cyclone

The air flow in a cyclone was considered as an isothermal incompressible flow and was modelled with the Large Eddy Simulation (LES) technique. The following governing filtered Navier–Stokes equations were solved

1 $$\begin{aligned}&\dfrac{\partial \overline{\overline{u_{i}}}}{\partial x_{i}}=0, \end{aligned}$$

2 $$\begin{aligned}&\dfrac{\partial \overline{\overline{u_{i}}}}{\partial t}+\overline{\overline{u_{j}}}\dfrac{\partial \overline{\overline{u_{i}}}}{\partial x_{j}}=-\dfrac{1}{\rho }\dfrac{\partial \overline{\overline{p}}}{\partial x_{i}}+\nu \dfrac{\partial ^2\overline{\overline{u_{i}}}}{\partial x_{j}\partial x_{j}}+\dfrac{\partial {\tau _{ij}^\textrm{sgs}}}{\partial x_{j}} \end{aligned}$$

where $\overline{\overline{u_{i}}}$ and $\overline{\overline{p}}$ are filtered air velocity and static pressure respectively; *t* is time; $\rho$ is air density; $\nu$ is air kinematic viscosity; and $\tau _{ij}^\textrm{sgs}$ is the SGS stress, which includes the effect of the small scales and is defined as:

3 $$\begin{aligned} \tau _{ij}^\textrm{sgs}=-\overline{\overline{u_{i}}\overline{u_{j}}}+\overline{\overline{u_{i}}}\overline{\overline{u_{j}}} . \end{aligned}$$

Applying the Boussinesq’s hypothesis, the SGS stress can be calculated as:

4 $$\begin{aligned}&\tau _{ij}^\textrm{sgs}-\frac{\delta }{3}\tau _{kk}^\textrm{sgs}=2\nu ^\textrm{sgs}\overline{\overline{S_{ij}}}, \end{aligned}$$

5 $$\begin{aligned}&\overline{\overline{S_{ij}}}=\frac{1}{2}\left( \dfrac{\partial \overline{\overline{u_{i}}}}{\partial x_{j}}+\dfrac{\partial \overline{\overline{u_{j}}}}{\partial x_{i}}\right) . \end{aligned}$$

where $\delta$ is the Kronecker delta ($\delta =1$ if $i=j$ and $\delta =0$ if $i\ne j$); $\nu ^\textrm{sgs}$ is subgrid-scale viscosity; and $\overline{\overline{S_{ij}}}$ is filtered strain rate tensor.

For subgrid-scale modelling a dynamic Smagorinsky–Lilly SGS model (Germano et al. 1991; Lilly 1992) was applied in this study. The choice of this model is explained in Misiulia et al. (2021) and the model is described in detail in Misiulia et al. (2022).

For discretisation of the governing equations the unbounded central difference advection scheme and the implicit time-stepping second order backward Euler transient scheme were applied. For pressure velocity coupling the fourth-order strategy similar to the one proposed by Rhie and Chow (Rhie and Chow 1983) and modified by Majumdar (Majumdar 1988) to remove the dependence of the steady-state solution on the time step was employed. The geometric shape functions and trilinear methods for interpolating nodal pressures to integration points for the pressure gradient term of the momentum equation and for interpolating nodal velocities to integration points for the velocity divergence term in the continuity equation were applied.

The advantage of using LES in simulating small-scale cyclones was proved by de Souza et al. (2012) who found that turbulence plays a very important role in the particle motion, as it causes the re-entrainment of particles that would be otherwise collected and consequently hinders the separation efficiency. Therefore, the appropriate modelling of not only the mean flow but also the instantaneous flow is crucial to the accurate prediction of the grade efficiency curve.

### Governing Equations for Particles

Particle transport was modelled by tracking particles through the flow, which was carried out by forming a set of ordinary differential equations in time for each particle, consisting of equations for position and velocity

6 $$\begin{aligned} \dfrac{dx_\textrm{p}}{dt}=u_\textrm{p}, \end{aligned}$$

where $u_\textrm{p}$ is a particle velocity; $x_\textrm{p}$ is a particle position, and

7 $$\begin{aligned} \frac{\pi }{6}d_\textrm{p}^{3}\rho _\textrm{p}\frac{d\overrightarrow{u_\textrm{p}}}{dt}=\overrightarrow{F_\textrm{D}}+\overrightarrow{F_\textrm{G}}, \end{aligned}$$

where $d_\textrm{p}$ is the particle diameter; $\rho _\textrm{p}$ is the particle density; $\overrightarrow{F_\textrm{D}}$ is a drag force acting on the particle; $\overrightarrow{F_\textrm{G}}$ is a net force due to gravity (which is equal to gravitational force minus buoyant force). Since the particle density is much higher than the air density, only the aerodynamic drag force and the net force due to gravity were taken into account.

The aerodynamic drag force was computed as

8 $$\begin{aligned} F_\textrm{D}=\dfrac{1}{2}C_\textrm{D}\rho \dfrac{\pi d_\textrm{p}^2}{4}\left\| u-u_\textrm{p}\right\| \left( u-u_\textrm{p}\right) , \end{aligned}$$

where the drag coefficient was calculated by using the Schiller Naumann correlation modified by a Cunningham correction factor

9 $$\begin{aligned} C_\textrm{D}=\dfrac{max\left[ \dfrac{24}{\textrm{Re}_\textrm{p}}\left( 1+0.15\,\textrm{Re}_\textrm{p}^{0.687}\right) , 0.44\right] }{C_\textrm{Cun}}, \end{aligned}$$

where particle Reynolds number

10 $$\begin{aligned} \textrm{Re}_\textrm{p}=\dfrac{d_\textrm{p}\left\| u-u_\textrm{p}\right\| }{\nu }. \end{aligned}$$

The Cunningham correction factor was calculated according to Davies (1945)

11 $$\begin{aligned} C_\textrm{Cun}=1+\dfrac{\lambda }{d_\textrm{p}}\left( 2.514+0.8e^{-0.55\dfrac{d_\textrm{p}}{\lambda }}\right) \end{aligned}$$

where $\lambda$ is mean free path of gas molecules ($\lambda =$  67.3 nm for air (Allen and Raabe 1982)).

For the integration of the ordinary differential Eqs. (6) and (7), the forward Euler integration scheme was used:

12 $$\begin{aligned} x_\textrm{p}^\textrm{n}=x_\textrm{p}^\textrm{o}+u_\textrm{p}^\textrm{o}\Delta t \end{aligned}$$

where superscripts ’o’ and ’n’ refer to old and new values respectively); and $u_\textrm{p}^\textrm{o}$ is the initial (old) particle velocity.

### Cyclone Geometry, Boundary Conditions and Numerical Settings

**Fig. 2:**
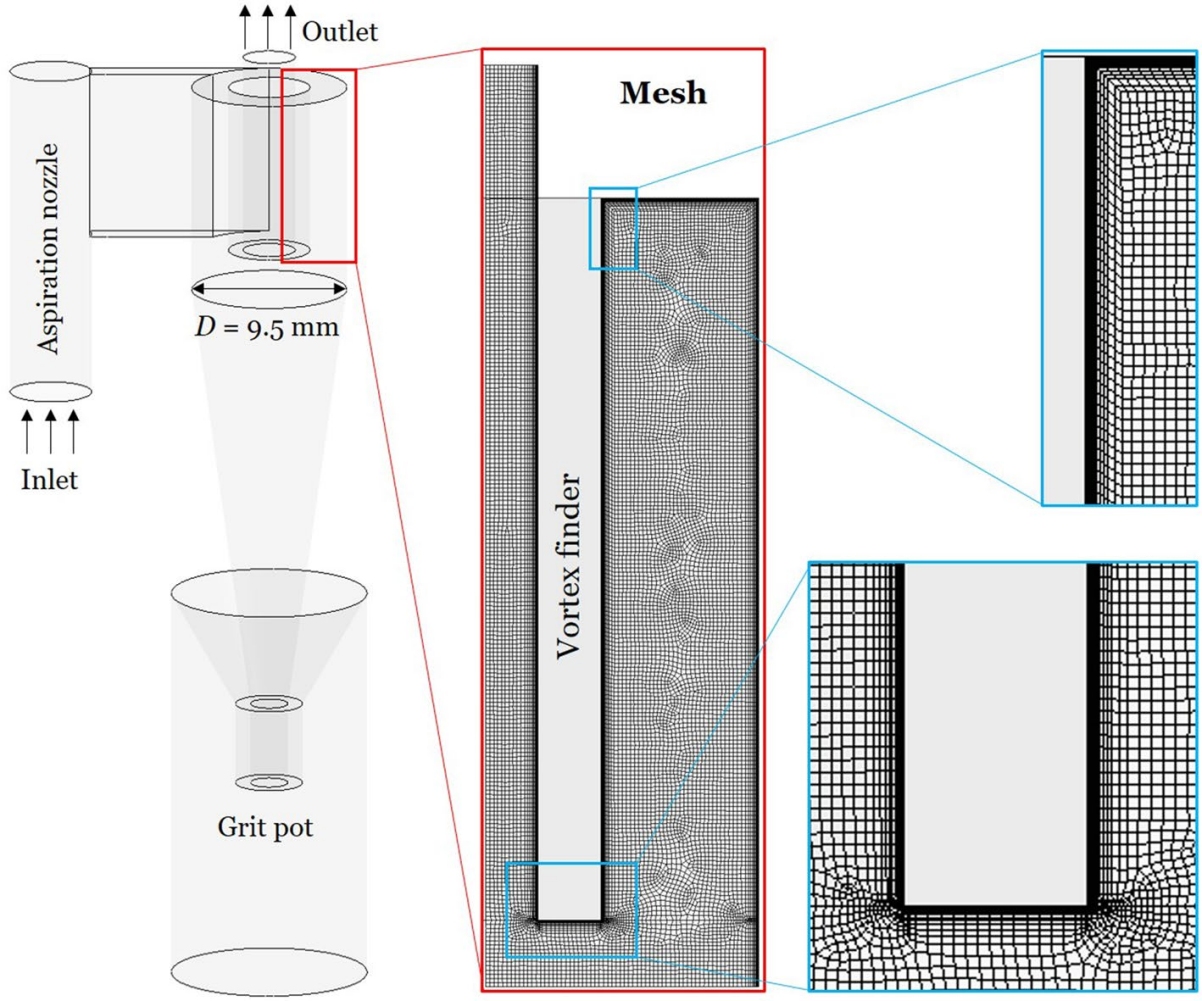
The investigated cyclone geometry and mesh

A 9.5 mm in diameter sampling cyclone of the HD design (Higgins and Dewell 1967) shown in Fig. 2 was investigated in this study. The geometrical dimensions of the cyclone can be found in Misiulia et al. (2021). This cyclone is used as a pre-separator during sampling by occupational hygienists of airborne industrial particles potentially causing occupational respiratory diseases (mainly solid particles, e.g. quartz dust and coal dust). The sample is collected onto a filter mounted downstream of the exit from the vortex finder. It has been manufactured in several commercial versions that are slightly different in minor aspects, e.g. BCIRA (British Cast Iron Association (no longer commercially available)), SIMPEDS (Safety in Mines Personal Dust Sampler (originally in metal but currently in plastic)), FSP2 and BGI4 (in both plastic and metal versions).

In order to create a realistic velocity profile, the total pressure was set at the inlet. At the outlet an opening boundary condition and atmospheric pressure were specified. Opening boundary condition implies that air can both leave and enter through the outlet, thus it allows the back flow at the outlet.

The numerical investigations were performed at inlet total pressure up to 800 Pa, which corresponded to flow rate through the cyclone up to 7.5 l per minute (LPM). Because of rather low Mach numbers even at the highest investigated flow rate, the air flow was considered incompressible with a density of 1.205 kg m$^{-3}$ and a dynamic viscosity of 1.831$\times 10^{-5}$ Pa s.

Cyclone walls were simulated as non-slip smooth walls. For a proper near-wall modelling, an automatic near-wall treatment method, developed by ANSYS CFX (ANSYS 2020), was applied. It automatically switches from wall-functions to a low-Re near wall formulation as the mesh is refined.

The numerical simulations consisted of two parts. First, simulations were performed without particles until the flow has reached statistically steady state. For that, flow rate through the cyclone as well as velocities at several points in the cyclone were monitored during simulations. When the flow has reached the fully developed state, the arithmetic averaging of the flow was initiated to obtain mean (time-averaged) parameters and spherical particles with a density of 1000 kg m$^{-3}$ were injected at the inlet with zero slip velocity. For a good statistics, 1000 particles of each diameter class were injected at the same time and tracked through the cyclone.

To obtain a so-called time-step independent solution of particle tracking, 100 integration steps per element were set in particle tracking integration (Misiulia et al. 2015).

In order to provide a constant particle concentration per unit volume and unit time across the inlet of the aspiration nozzle, particles were distributed at the inlet of the aspiration nozzle proportionally to the air velocity. For that, the time-averaged radial profile of the inlet velocity was analysed and described by the following expression (Misiulia et al. 2022)

13 $$\begin{aligned} \dfrac{\overline{u}}{\overline{u^\textrm{max}}}=\left( 1-m_1\left( \dfrac{r}{r_\textrm{as}}\right) ^{n_1}\right) \left( 1-\left( \dfrac{r}{r_\textrm{as}}\right) ^{n_2}\right) \end{aligned}$$

where *r* is radius; $r_\textrm{as}$ is radius of the aspiration nozzle; and $m_1$, $n_1$, $n_2$ are coefficients, determined as follows

14 $$\begin{aligned} m_1&=0.2592\,u_\textrm{as}^{-0.177}; \end{aligned}$$

15 $$\begin{aligned} n_1&=0.0201\,u_\textrm{as}^3-0.299\,u_\textrm{as}^2+1.684\,u_\textrm{as}+2.657; \end{aligned}$$

16 $$\begin{aligned} n_2&=0.3875\,u_\textrm{as}^3-6.055\,u_\textrm{as}^2+43.15\,u_\textrm{as}+12.44. \end{aligned}$$

where $u_\textrm{as}$ is the area-averaged mean air velocity in the aspiration nozzle, computed as the volumetric flow rate divided by the cross-sectional area of the aspiration nozzle

17 $$\begin{aligned} {u}_\textrm{as}=\frac{Q}{0.25\pi d_\textrm{as}^2} \end{aligned}$$

where *Q* is a volumetric flow rate through a cyclone; $d_\textrm{as}$ is the internal diameter of an aspiration nozzle; and $\mu$ is a dynamic viscosity.

The Eqs. (13–16) were used for setting the particle locations at the inlet. The model is based on the assumption that the particle concentration (per particle size) is identical in all air “parcels” aspirated. This is an ideal case and not realistic when the settling speed of a particle size is high in relation to the average air aspiration velocity. See the discussion in Misiulia et al. (2022).

The standard particle-wall interaction model was applied on walls. After collision with a cyclone wall a particle sticks to it resembling the behaviour of droplets. The action of particles when they hit a wall was described by the parallel and perpendicular restitution coefficients, which both were set to zero. The basis for choosing this value is the results from (Zhu and Lee (1999)). In a cyclone with a cylinder diameter of 30.5 mm, operating at inlet velocities of 17 and 23 m/s, they found no difference in measured separation curves determined with either solid or liquid test particles, respectively. The separation of liquid particles might be much more complicated, see e.g. the papers by Gao et al. (2013) and Wang et al. (2017). However, the authors did not go into this as for these small cyclones operated at low inlet velocities, measured separation efficiencies are almost identical for solid and liquid particles (as referred to in the authors’ paper Misiulia et al. 2022).

Cyclone samplers operate at very low particle concentrations therefore the effects of particles on the flow field was neglected.

## Mesh Independence Study and Validation

In CFD simulation, the results depend on the computational mesh, i.e. the finer the mesh is, the higher is the accuracy. In order to ensure that all the features of turbulence are predicted correctly, a finer mesh is required for larger Reynolds number. Based on that, the grid independence study was performed at the largest investigated flow rate (i.e. at the largest Reynolds number).

The effects of the mesh resolution on the cyclone pressure drop and separation efficiency, as well as on the flow field in the cyclone body was performed and described in detail in Misiulia et al. (2022). In this work, a grid independence study was performed to reveal the effects of the mesh resolution on the lip flow in a cyclone.

For the inlet pressure of 800 Pa, which corresponds to the highest investigated flow rate, several meshes consisting of (0.92–9.98) million hexahedron elements were generated and tested. The mesh near cyclone walls was refined with inflation layers (Fig. 2) to provide a proper flow resolution near walls. The highest values of the wall parameter $y^+$ were determined on the vortex finder wall and even there the maximum value of the area-averaged $y^+$ was below 1.0 in all simulations. Each mesh was characterised by the mean cell size

18 $$\begin{aligned} \langle \varDelta \rangle =\root 3 \of {\dfrac{V_\textrm{c}}{N}} \end{aligned}$$

where $V_\textrm{c}$ is a cyclone volume; *N* is a number of mesh elements (cells).

To evaluate accurately the numerical uncertainties in the computational results, the grid convergence index (*GCI*)—proposed by Roache (1998)—was applied. It is based upon a grid refinement error estimator derived from the theory of the generalized Richardson extrapolation. The *GCI* is a measure of how far the computed value deviates from the value of the asymptotic numerical value, i.e. it indicates how much the solution would change with a further refinement of the grid.

**Fig. 3:**
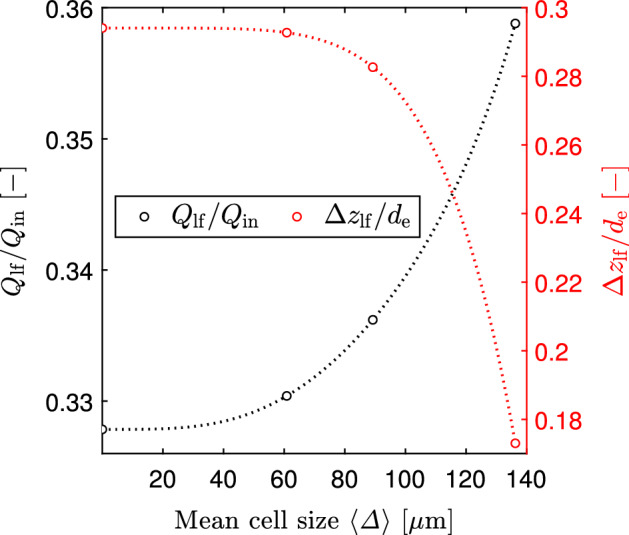
Qualitative representation of the grid independence study for the lip flow at flow rate 7.54 LPM

The *GCI* calculations were performed for the dimensionless lip flow rate normalised by the inlet flow rate ($Q_\textrm{lf}/Q_\textrm{in}$) and for the dimensionless thickness of the lip flow normalised by the internal diameter of the vortex finder ($\Delta z_\textrm{lf}/d_\textrm{e}$) according to the equations described in Misiulia et al. (2021). The method for the determination of the lip flow and its thickness is described in next section. Qualitative representation of the grid independence study for the lip flow is given in Fig. 3.

Figure 3 shows that both solutions (for the lip flow and the thickness of the lip flow region) are in the asymptotic range and converges monotonically. The mesh with a mean cell size below 70 $\mu$m provides the lip flow with an error less than 1%. Therefore, the mesh with a mean cell size of 66.6 $\mu$m was used in all simulations.

The validation of the simulations was done earlier (Misiulia et al. 2021, 2022) by comparing the simulated cyclone performance characteristics with the experimental measurements. The simulated pressure drop, cut-size and slope of the penetration curve agreed well with the experimental data. A detailed information can be found in the author’s previous papers (Misiulia et al. 2021, 2022).

Based on the given grid independence study as well as on the one earlier (Misiulia et al. 2022) performed at the largest investigated flow rate the authors infer that the simulation can be trusted in the whole investigated range of pressure drops, i.e. at flow rates up to 7.5 LPM.

## Results and Discussion

### Secondary Inward Radial Flow Along the Cyclone Lid

First, the secondary inward radial flow along the cyclone lid was analysed. For that, the omnidirectional (angle-averaged) time-averaged radial velocity was calculated at different radial positions $\Delta r$ determined as

19 $$\begin{aligned} \Delta r=\dfrac{r-R_\textrm{vf}}{R-R_\textrm{vf}} \end{aligned}$$

where *r* is a current radial position; $R_\textrm{vf}$ is the external radius of the vortex finder; and *R* is the internal cyclone radius. $\Delta r$ of 0 corresponds to the external radius of the vortex finder, whereas $\Delta r$ of 1 corresponds to the cyclone wall.

The radial inward flow rate was calculated by integration of the omnidirectional time-averaged radial velocity over the axial displacement $\Delta z$

20 $$\begin{aligned} Q_\textrm{r}=u_\textrm{r}2\pi r \Delta z \end{aligned}$$

**Fig. 4:**
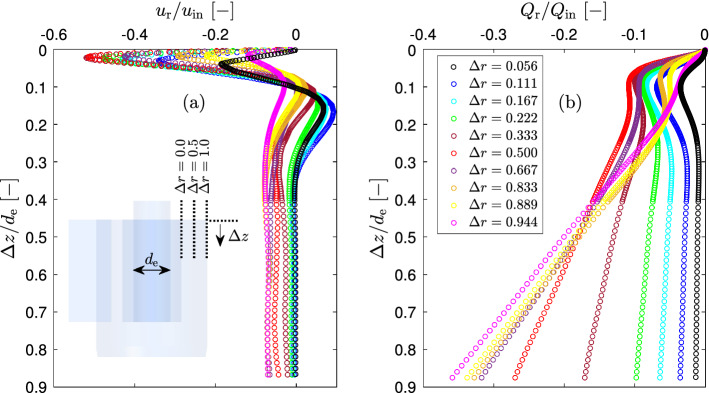
Omnidirectional time-averaged radial velocity **a** and radial flow rate **b** at different radial positions $\Delta r$ and at flow rate 7.54 LPM (sign “−” indicates that the radial flow direction is inward, i.e. it is in the direction of a decreasing cyclone radius)

Figure 4a shows, that there is indeed a strong inward radial flow in the vicinity of the cyclone lid. The radial velocities increase in the normal direction from the lid and reaches its maximum value (a bit larger than a half of ) at the distance from the lid about of 2.3% of the internal vortex finder diameter and at the middle radial position. At larger axial distances from the cyclone lid the radial velocity decreases to zero or even below and then remains fairly constant slightly above zero.

**Fig. 5:**
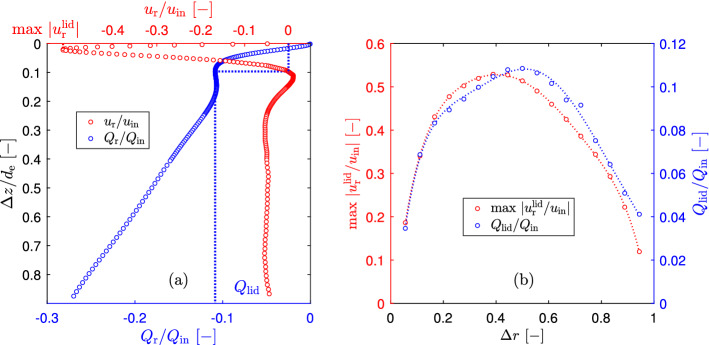
The method of determination the secondary inward radial flow along the cyclone lid **a** and maximal omnidirectional time-averaged inward radial velocity and radial flow rate at different radial positions **b** at flow rate 7.54 LPM (sign “−” indicates that the radial flow direction is inward, i.e. it is in the direction of a decreasing cyclone radius)

The inward radial flow rate (Fig. 4b) sharply increases with the increasing axial distance from the cyclone lid $\Delta z$. Practically at all radial positions there is an inflection point in the radial flow rate at $\Delta z/d_\textrm{e}\approx 0.1$. This inflection point is determined by the axial position where the radial velocity changes its direction from inward to outward. The secondary flow along the cyclone lid at each radial position was determined at this point. Figure 5a illustrates the method of determination the secondary flow along the cyclone lid at radial position of $\Delta r=0.5$ and at flow rate 7.54 LPM.

The secondary inward radial flow rate and the maximum inward radial velocity at different radial positions at a flow rate of 7.54 LPM are plotted in Fig. 5b. A shape of both curves represents a parabola, which opens downwards. This agrees with the radial profile of the secondary flow along the cyclone lid theoretically calculated by Ebert (1967). The maximum value of the inward radial flow near the cyclone lid corresponds to the central radial position between the vortex finder external wall and the cyclone wall ($\Delta r=0.5$) and equals 10.9% of the inlet flow. The maximum inward radial velocity along the cyclone lid is created at a radial position, which is a bit displaced to the vortex finder ($\Delta r=0.389$) and reaches 52.9% of the inlet velocity.

**Fig. 6:**
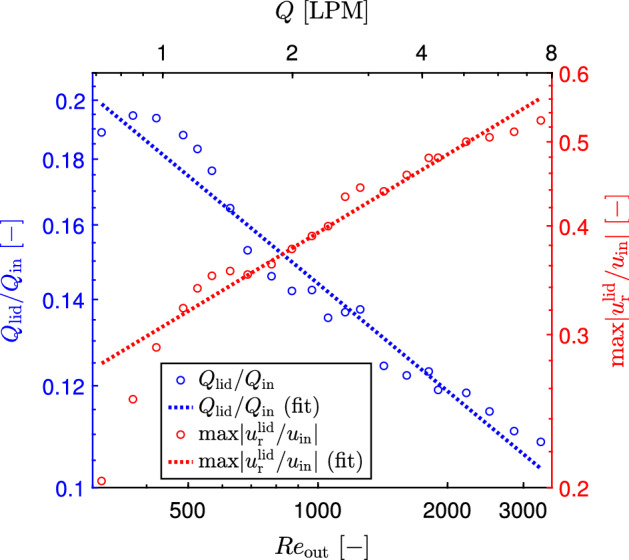
Maximal omnidirectional time-averaged radial velocity and radial flow rate along the cyclone lid, as a function of the flow Reynolds number in vortex finder

Similarly, the mentioned radial velocity and radial flow rate along the cyclone lid were determined for all other flow rates and the final results, presented as the maximum dimensionless omnidirectional time-averaged inward radial velocity and radial flow rate (secondary radial flow rate along the cyclone lid) are presented in Fig. 6.

The maximum inward radial velocity along the cyclone lid increases with the increasing flow rate (Reynolds number). In contrast to that, the secondary inward radial flow along the cyclone lid decreases from circa 20% down to 11% with increasing Reynolds number from 300 to 3300 (Fig. 6). This is explained by the reduction of the thickness of this secondary flow, which is much more significant than the increase in radial velocity. The secondary radial flow along the lid somehow agrees with the experimental finding of Trefz (1992), who stated the mean value of 10–15%. However they obtained these values in an industrial-scale cyclone at much larger Reynolds numbers. The dependence of these two characteristics on the Reynolds number in the range 300–3300 can be described by the following equations

21 $$\begin{aligned}&Q_\textrm{lid}/Q_\textrm{in}=0.983\,\textrm{Re}_\textrm{out}^{-0.278} \end{aligned}$$

22 $$\begin{aligned}&\textrm{max} \left\| u_\textrm{r}^\textrm{lid}/u_\textrm{in}\right\| =0.05\,\textrm{Re}_\textrm{out}^{0.3} \end{aligned}$$

The coefficients of determination ($R^2$) for Eqs. (21) and (22) are 0.956 and 0.948 respectively.

The Reynolds number in the vortex finder is calculated as

23 $$\begin{aligned} \textrm{Re}_\textrm{out}=\frac{{u}_\textrm{out} d_\textrm{e}\rho }{\mu } \end{aligned}$$

where $u_\textrm{out}$ is the mean axial velocity in the vortex finder

24 $$\begin{aligned} u_\textrm{out}=\frac{Q}{0.25\pi d_\textrm{e}^2} \end{aligned}$$

where $d_\textrm{e}$ is the vortex finder internal diameter.

### Secondary Downward Axial Flow Along the External Surface of the Vortex Finder

**Fig. 7:**
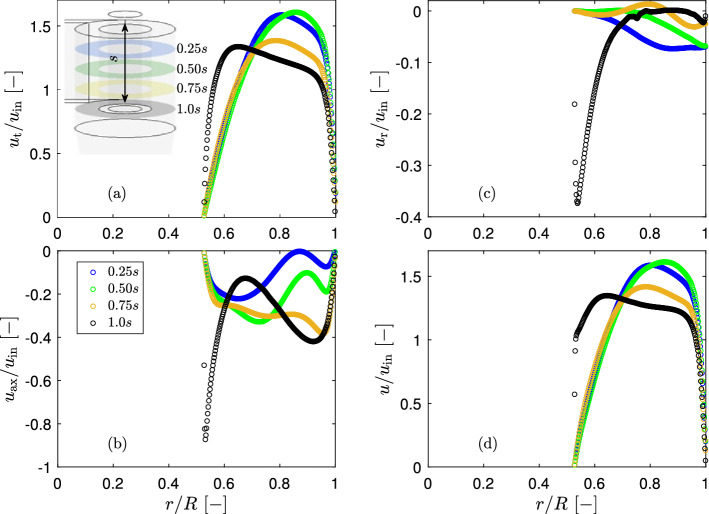
Omnidirectional time-averaged tangential velocity **a**, axial velocity **b**, radial velocity **c** and full velocity **d** in the annular region at flow rate 7.54 LPM

In order to determine the downward axial flow along the external wall of the vortex finder, the flow field in the annular region between the vortex finder and cyclone wall was investigated. The radial profile of three omnidirectional time-averaged velocity components (tangential, axial and radial) as well as full velocity was calculated at four axial planes along the vortex finder, which were displaced from the cyclone lid by 25, 50, 75 and 100% of the vortex finder depth *s* (Fig. 7).

The tangential velocity reduces with increasing displacement from the cyclone lid down to axial position of $\Delta z=0.75s$. At the lowest part of the annular region (at $\Delta z=\,$(0.75–1.0)*s*) the radial profile of the tangential velocity changes. At radial position $r/R=$ 0.5–0.7 tangential velocity sharply increase while at $r/R=$ 0.7–1.0 it slowly decreases.

Since the tangential velocity is much higher than the axial and radial velocities, the radial profile of the full velocity does not deviate significantly from the tangential velocity.

**Fig. 8:**
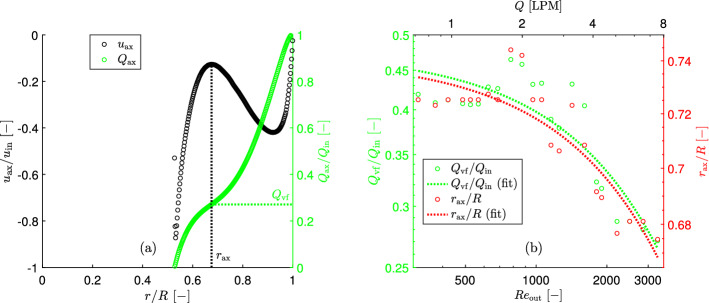
The method of determination **a** and the secondary downward axial flow along the external surface of the vortex finder **b** at $\Delta z/d_\textrm{e}=1$ and flow rate 7.54 LPM

The most interesting are the axial and radial velocities. At $\Delta z=$(0.75–1.0)*s* the axial velocities along the external surface of the vortex finder only slightly increase and the radial velocities are very small and almost constant there. In order to determine the secondary downward axial flow along the external surface of the vortex finder a specific criterion should be determined in order to distinguish this secondary flow from the main downward axial flow. However at the vortex finder inlet plane ($\Delta z=1.0s$), there is a high gradient and peak value in both axial and radial velocities in the vicinity to the vortex finder wall. That indicates the lip flow. Therefore, the radial profile of axial velocity at the vortex finder inlet plane was analysed in detail. It shows two axial flows, the main flow at $r/R>0.675$ and the secondary axial flow at $r/R<0.675$, which equals to 27.1% of the inlet flow (Fig. 8a). The latter was calculated by integration the radial profile of omni-directional time-averaged axial velocity over the radius

25 $$\begin{aligned} Q_\textrm{ax}=\int u_\textrm{r}rdr \end{aligned}$$

Similarly, the secondary downward axial flow at the vortex finder inlet plane $Q_\textrm{vf}$ and the corresponding radial position $r_\textrm{ax}$ were calculated for all flow rates and are shown in Fig. 8b.

The maximum secondary axial flow along the vortex finder (i.e. at the vortex finder inlet plane) and the corresponding radial position can be determined according to the following exponential equations

26 $$\begin{aligned}&Q_\textrm{vf}/Q_\textrm{in}=0.475\,exp (-\,0.000178\,\textrm{Re}_\textrm{out}) \end{aligned}$$

27 $$\begin{aligned}&r_\textrm{ax}/R=0.741\,exp (-\,0.0000313\,\textrm{Re}_\textrm{out}) \end{aligned}$$

This secondary flow is much larger than the secondary flow along the lid. The cause for this higher flow is that it also contains air that flows downward not close to the external surface of the vortex finder, but instead flows down more “midway” in the annular space.

### Inward Radial Flow Below the Vortex Finder

**Fig. 9:**
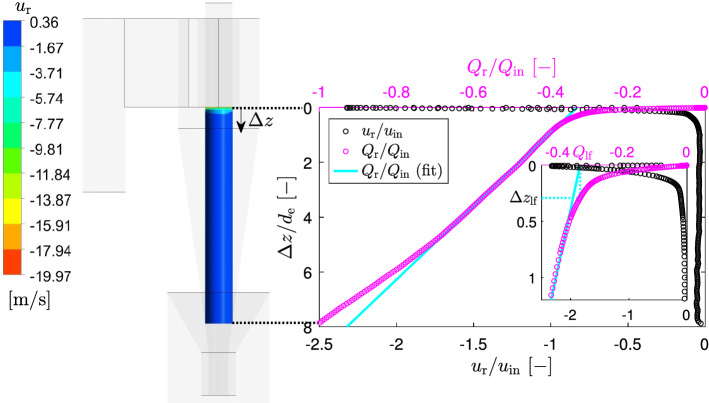
Omnidirectional time-averaged radial velocity and radial flow rate along the control surface at flow rate 7.54 LPM (insert shows the lip flow and its thickness)

Finally, the inward radial flow along the control surface (a cylindrical surface below the vortex finder with a diameter equal to the internal diameter of the vortex finder) was analysed. For all flow rates, the inward radial velocity was calculated at the control surface at different axial position and the radial inward radial flow rate was calculated according to Eq. (20). As an example, the radial inward flow rate for an inlet flow rate of 7.54 LPM is shown in Fig. 9.

There is a very strong radial inward flow just below the vortex finder, which is referred to lip flow. The radial velocity there exceed the inlet velocity by approximately 2.35 times.

The following method was applied to determine the lip flow and its thickness. In most of cyclone models it is assumed that the radial velocity is constant at the control surface, i.e. the radial flow rate linearly increases with increasing control surface length. This principle was used in our method, i.e. the lip flow was determined by linear extrapolation of the radial flow rate. The thickness of the lip flow region was taken at the corresponding value of the lip flow (see Fig. 9). This method gives the lip flow of 33.04% of the inlet flow and the thickness $\Delta z_\textrm{lf}/d_\textrm{e}=0.2927$. The effect of mesh resolution on these two parameters of the lip flow are presented in Fig. 3.

**Fig. 10:**
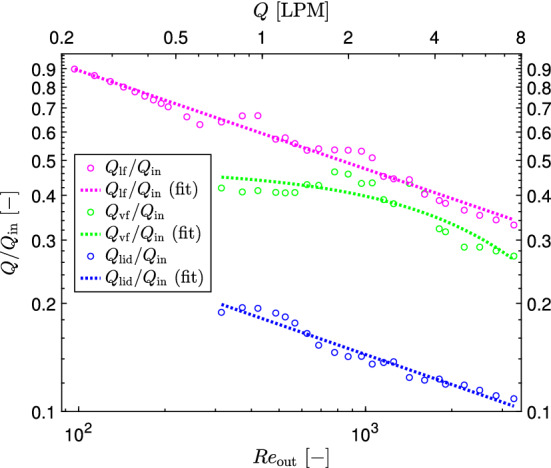
Secondary radial flow along the cyclone lid, secondary axial flow along the external surface of the vortex finder at $\Delta z/d_\textrm{e}=1$, and the lip flow below the vortex finder

Using this method the lip flow was determined at all flow rates and plotted in Fig. 10 together with the secondary inward radial flow along the cyclone lid and secondary downward axial flow along the external surface of the vortex finder.

Similarly as two above-mentioned secondary flows, the lip flow decreases with the increasing Reynolds number. It can be quite well approximated by a power model

28 $$\begin{aligned} Q_\textrm{lf}/Q_\textrm{in}=3.143\,\textrm{Re}_\textrm{out}^{-0.274} \end{aligned}$$

Interesting, there is a strong correlation between the secondary flow along the lid and the lip flow. Both these flows are described by a power model with almost the same power factor, $-\,0.278$ (Eq. 21) and $-\,0.274$ (Eq. 28). It means that the ratio of the lip flow to the secondary flow along the lid is almost independent of the Reynolds number and equals approximately 3.2. Possibly at much higher Reynolds numbers, corresponding to industrial-scale cyclones, the lip flow will reduce down to 10–15% as it was found by Trefz (1992) at $\textrm{Re}_\textrm{out}=$ 190000–260000. However for small-scale cyclones, the Muschelknautz model requires some amendments.

**Fig. 11:**
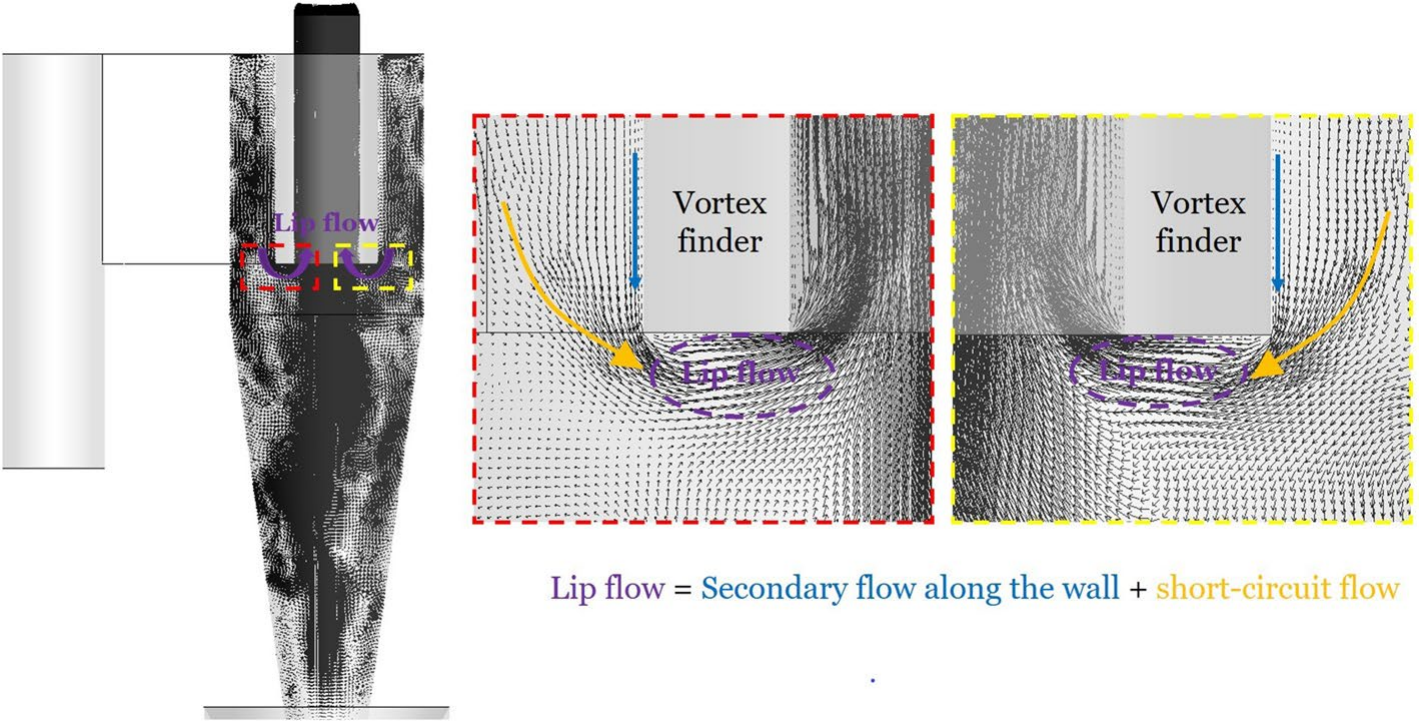
Velocity vector plot in the cyclone

The velocity vector plot (Fig. 11) shows that the lip flow consists of not only the secondary flow along the outer surface of the vortex finder but also of the short-circuit flow. A certain amount of the flow entering the cyclone body seeks to leave the cyclone at the shortest way, i.e. just below the vortex finder. This flow is referred to as short-circuit flow. This flow is approximately by 2.2 times larger that the secondary flow along the cyclone lid.

**Fig. 12:**
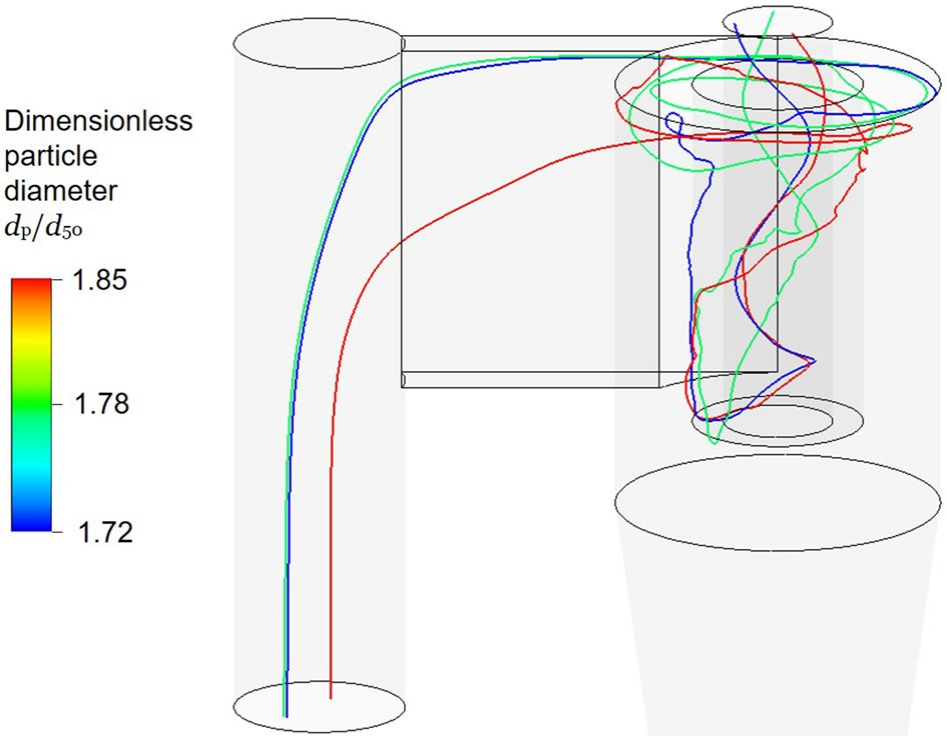
Trajectory of particles entrained by the lip flow in a cyclone

Why should one care about the lip flow in a cyclone? Because it deteriorates the separation capability of a cyclone. Figure 12 illustrates that even rather large particles can be entrained by this flow and leave the cyclone whereas these particles in the main separation zone are separated with efficiency above 95%.

In addition to the lip flow, there are other mechanisms that prevent particles from being separated in cyclones, for instance, they can be re-entrained along the inner vortex as it was demonstrated by de Souza et al. (2012). On the relevant boundary between the exiting upward flow (inner part of the vortex) and the downward flow (outer part of the vortex) in the cyclone separation chamber a particle motion is governed by the sum of the following radial forces:

- Temporal average of drag minus centrifugal force, and;
- Particle turbulent diffusion.

If one assumes that the particle concentration is always lower in the inner vortex than in the cyclone separation chamber, the turbulent diffusion will tend to move particles into the exiting inner vortex, i.e. reducing the separation efficiency of the cyclone. Possibly, to compare particle diffusion for a range of flow rates for which relevant particle sizes will differ, a Peclet number introduced by Salcedo and Coelho (1999) and a model of (Mothes and Löffler (1988)) can be used.

## Conclusion

Particle-laden air flow in a 9.5 mm in diameter HD cyclone has been numerically investigated for a wide range of flow rates with Large Eddy Simulation. Three secondary flows, inward radial flow along the cyclone lid, downward axial flow along the external surface of the vortex finder, and the inward radial flow below the vortex finder have been investigated in detail. The following conclusions can be drawn:

- The lip flow and the secondary flow along the cyclone lid decrease with the increasing flow rate (Reynolds number). This can be an additional explanation of why the particle separation efficiency of a cyclone increases with the increasing inlet velocities;
- The lip flow is much larger that the secondary flow along the lid because of the short-circuit flow, which constitutes the main contribution to the lip flow;
- Ratio of the lip flow to the secondary flow along the lid is practically independent of the Reynolds number and equals approximately 3.2;
- The lip flow in small-scale cyclones is much larger than 10% applied in the widely used Muschelknautz method of modelling. Relatively large lip flow can explain the non-applicability of the Muschelknautz model for small-scale cyclones. The latter needs to be refined in this regard.

Future extension of this work is to study the lip flow in cyclone separators at much wider range of Reynolds numbers. This will be realised by investigating several cyclones of different sizes at various inlet velocities.

## List of Symbols

$C_\textrm{Cun}$: Cunningham correction factor (–)
$$C_\textrm{D}$$: Drag coefficient (–)
$$F_\textrm{D}$$: Drag force (N)
$F_\textrm{G}$: Net force due to gravity (N)
*N*: Number of mesh cells (–)
*Q*: Volumetric flow rate (M$^3$ s$^{-1}$)
$Q_\textrm{in}$: Inlet flow rate (M$^3$ s$^{-1}$)
$$Q_\textrm{lf}$$: Lip flow (M$$^3$$ s$$^{-1}$$)
$Q_\textrm{lid}$: Maximum secondary inward radial flow along the cyclone lid (M$^3$ s$^{-1}$)
$Q_\textrm{r}$: Radial flow rate (M$^3$ s$^{-1}$)
$Q_\textrm{vf}$: Maximum secondary downward axial flow along the external wall of the vortex finder (M$^3$ s$^{-1}$)
*R*: Internal radius of a cyclone body (M)
$R_\textrm{vf}$: External radius of a vortex finder (M)
$\textrm{Re}_\textrm{out}$: Reynolds number at the outlet (–)
$\textrm{Re}_\textrm{p}$: Particle Reynolds number (–)
$S_{ij}$: Strain rate tensor (S$^{-1}$)
$$V_\textrm{c}$$: Cyclone volume (M$$^3$$)
$d_\textrm{as}$: Internal diameter of the aspiration nozzle (M)
$d_\textrm{e}$: Vortex finder internal diameter (M)
$$d_\textrm{p}$$: Particle diameter (M)
*p*: Pressure (Pa)
*r*: Radius (M)
$r_\textrm{ax}$: Radius that splits the axial flow into the main flow and secondary flow at the vortex finder plane lid (M)
*s*: Vortex finder depth (M)
*t*: Time (S)
*u*: Velocity (M s$$^{-1}$$)
$u_\textrm{as}$: Area-averaged axial velocity in the aspiration nozzle (M s$^{-1}$)
$$u_\textrm{ax}$$: Axial velocity (M s$$^{-1}$$)
$u_\textrm{in}$: Area-averaged air velocity in a rectangular cyclone inlet (M s$^{-1}$)
$$u_\textrm{p}$$: Particle velocity (M s$$^{-1}$$)
$$u_\textrm{r}$$: Radial velocity (M s$$^{-1}$$)
$u_\textrm{r}^\textrm{lid}$: Radial velocity along the cyclone lid (M s$^{-1}$)
$$u_\textrm{t}$$: Tangential velocity (M s$$^{-1}$$)
$$y^+$$: Wall parameter (–)
$\Delta z_\textrm{lf}$: Thickness of the lip flow (M)

## Greek Letters

$$\delta$$: Kronecker delta (–)
$\varDelta$: Grid filter width (cell size) (M)
$\lambda$: Mean free path of gas molecules (M)
$\nu$: Air kinematic viscosity (M$^2$ s$^{-1}$)
$$\nu ^\textrm{sgs}$$: SGS viscosity (M$$^2$$ s$$^{-1}$$)
$\rho$: Air density (Kg m$^{-3}$)
$\rho _\textrm{p}$: Particle density (Kg m$^{-3}$)
$\tau _{ij}^\textrm{sgs}$: SGS stress at scale $\varDelta$ (M$^2$ s$^{-2}$)
$\overline{\overline{~~}}$: Filtered with a grid filter $\varDelta$
$$\langle ~\rangle$$: Volume-averaged

## Abbreviations

CFD: Computational fluid dynamics
GCI: Grid convergence index
HD: Higgins-Dewell
LDA: Laser doppler anemometry
LES: Large eddy simulation
LPM: Litre per minute

## References

[CR1] Allen MD, Raabe OG (1982). Re-evaluation of Millikan’s oil drop data for the motion of small particles in air. J. Aerosol Sci..

[CR2] ANSYS. Ansys CFX-Solver Theory Guide. Release 2020R2. Canonsburg, Pennsylvania: ANSYS, Inc (2020)

[CR3] Chia PY, Coleman KK, Tan YK, Ong SWX, Gum M, Lau SK, Milton DK (2020). Detection of air and surface contamination by SARS-CoV-2 in hospital rooms of infected patients. Nat. Commun..

[CR4] Davies CN (1945). Definitive equations for the fluid resistance of spheres. Proc. Phys. Soc..

[CR5] de Souza FJ, de Vasconcelos Salvo R, de Moro Martins DA (2012). Large eddy simulation of the gas-particle flow in cyclone separators. Sep. Purif. Technol..

[CR6] Ebert F (1967). Berechnung rotationssymmetrischer, turbulenter Grenzschichten mit Sekundärströmung.

[CR7] Gao X, Chen J, Feng J, Peng X (2013). Numerical and experimental investigations of the effects of the breakup of oil droplets on the performance of oil-gas cyclone separators in oil-injected compressor systems. Int. J. Refrig..

[CR8] Germano M, Piomelli U, Moin P, Cabot WH (1991). A dynamic subgridscale eddy viscosity model. Phys. Fluids A Fluid Dyn..

[CR9] Guo Z-D, Wang Z-Y, Zhang S-F, Li X, Li L, Li C, Chi X-Y (2020). Aerosol and surface distribution of severe acute respiratory syndrome coronavirus 2 in hospital wards Wuhan China. Emerg Infect Diseases.

[CR10] Higgins RI, Dewell P, Davies CN (1967). A gravimetric size-selecting personal dust sampler. Inhaled Particles and Vapours.

[CR11] Hoffmann AC, Stein LE (2008). Gas Cyclones and Swirl Tubes: Principles, Design and Operation.

[CR12] Hoffmann, D.: Sekundärströmungen im Tauchrohrbereich eines Zyklons und ihs Einflluß auf die Partikelabscheidung. PhD thesis. Technische Universität Graz (1998)

[CR13] Lilly DK (1992). A proposed modification of the germano subgrid-scale closure method. Phys. Fluids A Fluid Dyn..

[CR14] Majumdar S (1988). Role of underrelaxation in momentum interpolation for calculation of flow with nonstaggered grids. Numer. Heat Transf..

[CR15] Misiulia D, Andersson AG, Lundström TS (2015). Computational investigation of an industrial cyclone separator with helical-roof inlet. Chem. Eng. Technol..

[CR16] Misiulia D, Lidén G, Antonyuk S (2021). Evolution of turbulent swirling flow in a small-scale cyclone with increasing flow rate: A LES study. Flow Turbul. Combust..

[CR17] Misiulia D, Lidén G, Antonyuk S (2022). Performance characteristics of a small scale cyclone separator operated in different flow regimes. J. Aerosol Sci..

[CR18] Mothes H, Löffler F (1988). Prediction of particle removal in cyclone separator. Int. Chem. Eng.

[CR19] Muschelknautz, U.: L3.4 Zyklone zum Abscheiden fester Partikel aus Gasen. VDI-Wärmeatlas, pp. 1599–1617. Springer (2019)

[CR20] Rahmani AR, Leili M, Azarian G, Poormohammadi A (2020). Sampling and detection of corona viruses in air: A mini review. Sci. Total Environ..

[CR21] Rhie CM, Chow W-L (1983). Numerical study of the turbulent flow past an airfoil with trailing edge separation. AIAA J..

[CR22] Roache PJ (1998). Verification and Validation in Computational Science and Engineering.

[CR23] Salcedo RL, Coelho MA (1999). Turbulent dispersion coefficients in cyclone flow: An empirical approach. Can. J. Chem. Eng..

[CR24] Trefz, M.: Die verschiedenen Abscheidevorgänge im höher und hoch beladenen Gaszyklon unter besonderer Berücksichtigung der Sekundärströmung. Fortschr.-Ber. VDI Reihe 3 Nr. 295 (1992)

[CR25] Wang L, Feng J, Gao X, Peng X (2017). Investigation on the oil-gas separation efficiency considering oil droplets breakup and collision in a swirling flow. Chem. Eng. Res. Design.

[CR26] Zhu Y, Lee KW (1999). Experimental study on small cyclones operating at high flowrates. J. Aerosol Sci..

